# Loss of Tmem106b is unable to ameliorate frontotemporal dementia-like phenotypes in an AAV mouse model of *C9ORF72*-repeat induced toxicity

**DOI:** 10.1186/s40478-018-0545-x

**Published:** 2018-05-31

**Authors:** Alexandra M. Nicholson, Xiaolai Zhou, Ralph B. Perkerson, Tammee M. Parsons, Jeannie Chew, Mieu Brooks, Mariely DeJesus-Hernandez, NiCole A. Finch, Billie J. Matchett, Aishe Kurti, Karen R. Jansen-West, Emilie Perkerson, Lillian Daughrity, Monica Castanedes-Casey, Linda Rousseau, Virginia Phillips, Fenghua Hu, Tania F. Gendron, Melissa E. Murray, Dennis W. Dickson, John D. Fryer, Leonard Petrucelli, Rosa Rademakers

**Affiliations:** 1 Department of Neuroscience, Mayo Clinic Jacksonville, 4500 San Pablo Road, Jacksonville, FL 32224 USA; 2 Department of Molecular Biology and Genetics, Weill Institute for Cell and Molecular Biology, Cornell University, 345 Weill Hall, Ithaca, NY 14853 USA

## Abstract

Loss-of-function mutations in progranulin (*GRN*) and a non-coding (GGGGCC)_n_ hexanucleotide repeat expansions in *C9ORF72* are the two most common genetic causes of frontotemporal lobar degeneration with aggregates of TAR DNA binding protein 43 (FTLD-TDP). *TMEM106B* encodes a type II transmembrane protein with unknown function. Genetic variants in *TMEM106B* associated with reduced TMEM106B levels have been identified as disease modifiers in individuals with *GRN* mutations and *C9ORF72* expansions. Recently, loss of Tmem106b has been reported to protect the FTLD-like phenotypes in *Grn−/−* mice. Here, we generated *Tmem106b−/−* mice and examined whether loss of Tmem106b could rescue FTLD-like phenotypes in an AAV mouse model of *C9ORF72*-repeat induced toxicity. Our results showed that neither partial nor complete loss of Tmem106b was able to rescue behavioral deficits induced by the expression of (GGGGCC)_66_ repeats (66R). Loss of Tmem106b also failed to ameliorate 66R-induced RNA foci, dipeptide repeat protein formation and pTDP-43 pathological burden. We further found that complete loss of Tmem106b increased astrogliosis, even in the absence of 66R, and failed to rescue 66R-induced neuronal cell loss, whereas partial loss of Tmem106b significantly rescued the neuronal cell loss but not neuroinflammation induced by 66R. Finally, we showed that overexpression of 66R did not alter expression of *Tmem106b* and other lysosomal genes in vivo, and subsequent analyses in vitro found that transiently knocking down *C9ORF72*, but not overexpression of 66R, significantly increased TMEM106B and other lysosomal proteins. In summary, reducing Tmem106b levels failed to rescue FTLD-like phenotypes in a mouse model mimicking the toxic gain-of-functions associated with overexpression of 66R. Combined with the observation that loss of C9ORF72 and not 66R overexpression was associated with increased levels of TMEM106B, this work suggests that the protective *TMEM106B* haplotype may exert its effect in expansion carriers by counteracting lysosomal dysfunction resulting from a loss of C9ORF72.

**Electronic supplementary material:**

The online version of this article (10.1186/s40478-018-0545-x) contains supplementary material, which is available to authorized users.

## Introduction

Frontotemporal dementia (FTD) is a devastating neurodegenerative disorder with initial symptoms occurring in the fifth or sixth decade of life. While most cases of FTD develop sporadically, 30–50% of FTD cases report a family history [23, 43, 44, 47, 61], in support of a strong genetic component to the disease. Two of the most common gene mutations found to cause FTD reside in the progranulin (*GRN*) and chromosome 9 open reading frame 72 (*C9ORF72*) genes [6, 14, 17, 45]. Causative *GRN* mutations leading to FTD include heterozygous missense, nonsense, or frameshift changes that most often lead to nonsense-mediated decay of the mutant mRNA and an associated loss of progranulin protein (PGRN). Individuals with *GRN* mutations invariably present with aggregates of the TAR DNA binding protein 43 (TDP-43) in affected brain regions, and are thus pathologically classified as FTLD-TDP [4, 36]. In *C9ORF72*, a non-coding (GGGGCC)_n_ hexanucleotide repeat expansion is responsible for up to 25% of familial and 5% of sporadic FTD patients [17, 45]. Extensive research has shown that the presence of these expanded repeats leads to multiple pathogenic mechanisms, including a loss of *C9ORF72* mRNA expression and toxic gain-of-functions resulting from nuclear RNA aggregates and dipeptide repeats proteins [5, 17, 33, 34, 45]. FTD patients with *C9ORF72* expansions also present with FTLD-TDP at autopsy, suggesting a potentially convergent disease mechanism between *GRN-* and *C9ORF72*-induced pathogenesis.

In 2010, a genome-wide association study (GWAS) identified genetic variants at the transmembrane protein 106 B (*TMEM106B*) gene locus as the first genetic modifiers of FTLD-TDP [59]. *TMEM106B* variants were found to be a modifier of disease risk in FTLD-TDP patients of unknown cause, and a modifier of disease penetrance and presentation in *GRN* mutation and *C9ORF72* expansion carriers [13, 19, 21, 30, 37, 58–60]. Specifically, in *C9ORF72* carriers, we showed that individuals who were also homozygous for the minor alleles at the associated *TMEM106B* variants were significantly protected from developing FTD but not amyotrophic lateral sclerosis (ALS) symptoms [18, 58], another common phenotypic presentation in *C9ORF72* expansion carriers.

The TMEM106B protein resides in lysosomal compartments where it might be involved in lysosomal function and/or trafficking [7, 11, 29, 50, 53]. Overexpression of TMEM106B results in abnormal lysosomal size, number, and acidification [7, 11]. Interestingly, recent studies determined that the protective *TMEM106B* variants are associated with reduced levels of TMEM106B [20, 37, 59], suggesting that lowering TMEM106B might be therapeutic in the context of FTD. In fact, lysosomal deficits observed in *Grn* knockout mice were recently rescued by loss of Tmem106b expression [26]. In this study, we aimed to examine whether loss of Tmem106b expression was able to rescue FTD-like behavioral and pathological features observed in an adeno-associated virus (AAV)-based mouse model mimicking the toxic gain-of-functions associated with overexpression of (GGGGCC)_66_ repeats.

## Methods

### Tmem106b knockout mice

Tmem106b knockout mice were generated at the Knockout Mouse Project (KOMP) Repository at the University of California, Davis using the PGS00041_A_C06 targeting vector and blastocyst injection of the targeted embryonic stem cell clone EPD0047_1_E02 generated from C57BL/6 N mice. This knock-in first strategy results in the insertion of a lacZ gene trap between the first two coding exons (exons 3 and 4) of the mouse *Tmem106b* gene. Cryopreserved sperm were purchased and used to inseminate oocytes obtained from 3-week-old C57BL/6N female mice (Harlan, Indianapolis, IN). Zygotes that reached the 2-cell-stage 24 h post insemination were surgically transferred into foster dams (Harlan). DNA obtained from subsequent pups was screened by multiplex polymerase chain reaction (PCR) for the presence of the NEO cassette before breeding as a colony founder (CSD-Tmem106b-F: 5’-TTCTCTCCATGTGCTGCATTATGAGC-3′; CSD-Neo-F: 5’-GGGATCTCATGCTGGAGTTCTTCG-3′; CDS-Tmem106b-ttR: 5’-ACGTGCTTCTCTCATCTACAGTTTTCC-3′). A *Tmem106b+/−* x *Tmem106+/−* breeding scheme was used to generate *Tmem106b +/+*, *+/−*, and *−/−* mice for the experiments. Both male and female mice of each *Tmem106b* genotype were used for all the experiments. All animal studies were approved by the Mayo Clinic Institutional Animal Care and Use Committee.

### Genotyping

Genomic DNA (gDNA) was extracted and PCR-amplified using the Phire Tissue Direct Master Mix kit (Thermo Scientific Inc., Waltham, MA) per the manufacturer’s instructions. Briefly, mouse hair follicles were digested in Dilution Buffer supplemented with DNARelease Additive for 2 min at room temperature, followed by 2 min incubation at 95 °C. Samples were briefly centrifuged and 1 μl of supernatant containing the gDNA was used for each PCR reaction. *Tmem106b* gene products were amplified using a multiplex PCR approach containing 0.8 μM of each forward primer (CSD-Tmem106b-F and CSD-Neo-F), 0.8 μM of reverse primer (CDS-Tmem106b-ttR), and Phire Tissue Direct PCR Master Mix (Thermo Scientific).

### Viral production and injections

Viruses were generated as previously described [12, 22, 54]. Briefly, (GGGGCC)_2_ (2R) or (GGGGCC)_66_ (66R) *C9ORF72* repeats were cloned into the pAM/CBA-pl-WPRE-BGH vector containing inverted repeats of serotype 2. AAV vectors containing the repeats were packaged into the serotype 9 type capsid by co-transfection with helper plasmids into HEK293T cells. The cells were harvested and lysed 2 days post transfection in the presence of 0.5% sodium deoxycholate and 50 U/ml Benzonase (Sigma Aldrich, St. Louis, MO) by freeze thawing. The virus was isolated using a discontinuous iodixanol gradient, and qPCR was used to determine the genomic titer of each virus. 2R and 66R AAV were diluted to 1_E_13 genomes/ml in sterile phosphate-buffered saline (PBS) before injection. Mouse pups underwent intracerebroventricular injections with virus at postnatal day 0 (P0) [10, 12, 25]. Pups were cryoanesthetized on ice and their heads were wiped with a sterile alcohol pad. Two microliters of virus were manually injected into each cerebral ventricle using a 32 gauge needle attached to a 10 μl syringe (Hamilton Company, Reno, NV). After injection, pups were warmed on a heating pad and placed back with the dam. All litters were injected within an 8-day timeframe and mice were aged to 12 months before assessing behavior and pathological manifestations. Small subsets of mice were harvested at 3 months of age to study *Tmem106b* expression and validate the model.

### Open field test

Mice were placed in a square, Perspex box (40x40x30cm, LxWxH) containing side-mounted photobeams placed 7.6 cm above the bottom of the box. Mice were allowed to move freely for 15 min, during which locomotor activity and anxiety measurements were taken. The Perspex box was illuminated by a light suspended over the chamber, and an overhead camera and AnyMaze software (Wood Dale, IL) were used to monitor mouse movement, such as time mobile, total distance traveled, and distance traveled in the outer and center zones. Mouse rearing was recorded by breaking of the photobeams.

### Conditional fear testing

Each mouse was placed in a sound-reducing chamber containing a grid floor capable of inducing an electric shock. An overhead camera and FreezeFrame software (Actimetrics, Wilmette, IL) were used to measure freezing. The mice were left undisturbed for the first 2 min of the test and baseline freezing was recorded. An 80-dB white noise was then administered for 30 s (conditioned stimulus; CS). During the last 2 s of the CS, a 0.5 mA foot shock was administered to the mouse (unconditioned stimulus; US). After 1 min, a second CS-US pair was given to the mouse and the mouse was removed from the chamber and placed in his/her home cage 30 s later. Each mouse was returned to the testing chamber 24 h later and freezing behavior was recorded for 5 min (context test). All mice were returned to their home cages and transferred to a different room with reduced light for at least 1 h. Contextual cues were changed by altering the environment, shape, and smell of the testing chamber, as well as by covering the chamber floor with opaque plastic. Each mouse was placed back into the test chamber and the auditory stimulus was presented. Freezing was recorded for 3 min (cued test). For both the context and cued tests, baseline freezing time was subtracted from the freezing time obtained during each test.

### Social interaction test

Each mouse was placed into a rectangular box subdivided into three chambers. Two larger chambers measured 17 × 40 cm with a smaller chamber of 5 × 40 cm in the middle. The three chambers were connected by an 8 × 5 cm opening to allow the mouse free access to all chambers. Two empty, inverted wire-mesh cylinders were placed in opposite corners of each large chamber. In the first trial, mice were placed in the box and allowed to explore the apparatus freely for 4 min before being placed into a temporary holding cage. Next, a probe mouse (matched for sex/strain) was placed in one of the cylinders for 3 min prior to reintroduction of the test mouse. An overhead camera and Anymaze software (Stoelting Co.) were used to monitor mouse interactions for 10 min. The time each test mouse spent in the area containing the cylinder with the probe mouse was used to determine sociability.

### Tissue harvests

Mice were subjected to carbon dioxide narcosis and body weight was obtained (SCALTEC SBA 53 scale; Denver Instrument, Bohemia, NY) before decapitation. Mouse blood was collected in tubes containing 1.6 mg/ml EDTA and placed on ice. Blood samples were centrifuged at 4 °C for 10 min at 5000 rpm, after which the resulting plasma supernatant was transferred to a new tube for storage at -80 °C until use. The brain was removed and its weight recorded (SCALTEC SBC 32 scale; Denver Instrument) before separating the hemispheres. Whole mouse brains from uninjected mice were either immediately dehydrated and flash frozen in a beaker of isopentane on dry ice or fixed for 24 h at 4 °C in 4% paraformaldehyde (PFA) prepared in PBS. For all injected mice, the left hemisphere was fixed in PFA at 4 °C for 48 h. The right hemisphere was immediately dehydrated and flash frozen in a beaker of isopentane on dry ice. Following PFA fixation, brain tissues were washed and stored in PBS at 4 °C until being embedded in paraffin wax.

### Cell culture and transfection

HeLa and U251 cells (ATCC, Manassas, VA) were cultured and maintained in Eagle’s Minimum Essential Medium (EMEM) supplemented with 10% FBS and 1% penicillin/streptavidin. All cell lines were maintained at 37 °C, 5% CO_2_. For overexpression studies, cells were transiently transfected with pAAV C9ORF72 2R or pAAV *C9ORF72* 66R using Lipofectamine 2000 (Invitrogen, Carlsbad, CA) by mixing DNAs with the transfection reagent in OptiMEM (Life Technologies, Carlsbad, CA, USA) according to the manufacturer’s protocol. For the siRNA knockdown experiments, HeLa and U251 cells were transfected with 20 nM of either negative control siRNA or siRNAs against human *C9ORF72* using Lipofectamine RNAiMAX Reagent (Life Technologies) according the manufacturer’s protocol. The control siRNA (5’-UGGUUUACAUGUCGACUAA-3′, D-001210-05) and human *C9ORF72* siRNA (5’-CAUAGAGUGUGUGUUGAUA-3′, J-013341-11) were purchased from Dharmacon (Lafayette, CO). Cells were harvested for protein extraction 48 and 72 h post transfection for overexpression and siRNA experiments, respectively.

### RNA and protein extraction

Frozen brain tissue was homogenized by sonication in tris-buffered saline (TBS) containing 2X protease and phosphatase inhibitors (Thermo Scientific). RNA was isolated from 75 μl of brain homogenate using the RNeasy Plus Mini Kit (Qiagen, #74136) according to the manufacturer’s instructions. Briefly, brain tissue was lysed using Buffer RLT containing β-ME, and then passed through the gDNA Eliminator column to remove DNA. The RNA containing flow-through was precipitated by 70% ethanol and passed through an RNeasy pink spin column. RNA was eluted from the column with RNase-free water. Protein was also isolated from 75 μl of brain homogenate by adding and equivalent volume of 2X Radioimmunoprecipitation Assay (RIPA) buffer (Boston BioProducts, Ashland, MA). For cell culture experiments, media was removed and RIPA buffer was added directly to PBS-rinsed cell culture wells. All RIPA samples were incubated on ice and centrifuged at 4 °C for 5 min at 6000 rpm to clear debris. Protein content in brain samples was measured in the supernatant using the bicinchoninic acid (BCA) assay (Thermo Fisher Scientific).

### Quantitative PCR

Brain RNA was reverse transcribed using the Superscript III complimentary DNA (cDNA) synthesis kit, random hexamers (Life Technologies), and an equal ratio of random hexamers and Oligo dT primers (Thermo Fisher Scientific). Real-time quantitative PCRs (qPCRs) were conducted using TaqMan gene expression assays and the QuantStudio 7 Flex Real-Time PCR System (Applied Biosystems, Foster City, CA). All probes were purchased from Life Technologies: *Tmem106a* (Mm01246747_m1), *Tmem106b* (Mm00510952_m1), *Tmem106c* (Mm01303550_m1), *Iba1* (*Aif1*; Mm00479862_g1), *Gfap* (Mm01253033_m1), and *Gapdh* (Mm99999915_g1).

### Western blotting

Protein samples were mixed with an equivalent volume of 2X Novex sample buffer (Life Technologies) supplemented to 5% β-mercaptoethanol. Proteins were denatured by incubating at room temperature for 30 min or by heating at 95 °C for 1–5 min before loading into SDS-polyacrylamide gels (Life Technologies). Proteins were transferred to Immobilon membranes (Millipore, Billerica, MA) and immunoblotted with the primary antibody at 4 °C overnight. The next day, blots were incubated with an HRP-conjugated secondary antibody (Promega, Madison, WI) and bands were detected by enhanced chemiluminescence using Western Lightning *Plus*-ECL reagents (Perkin Elmer, Waltham, MA). Primary antibodies included: rabbit anti-Tmem106b from Bethyl Laboratories (A303-439A), rabbit anti-Tmem106b generously shared and derived in the lab of Dr. Fenghua Hu, sheep anti-mouse progranulin (AF2557; R&D systems, Minneapolis, MN), goat anti-human progranulin (AF2420; R&D systems, Minneapolis, MN), mouse anti-Gapdh (H86504M; Meridian Life Sciences, Cincinnati, OH), mouse anti-Lamp1 (sc-20,011; Santa Cruz Biotechnology, Dallas, TX), rabbit anti-C9ORF72 (ABN1645; Millipore), mouse anti-HA (clone12CA5; #11583816001; Roche, Indianapolis, IN), and goat anti-Cathepsin-D (clone C-20; sc-6486; Santa Cruz Biotechnology, Dallas, TX). Bands of Western blots were quantified using Image J (NIH, Bethesda, MD).

### Poly(GP) immunoassay

Poly(GP) protein levels were measured in 10 μg of protein in duplicate from mouse brain lysates using a sandwich immunoassay utilizing MesoScale Discovery (MSD) technology as previously described [12, 22]. Serial dilutions of recombinant (GP)_8_ were used as a standard curve. Response values were measured using the MSD QUICKPLEX SQ120 and are defined as the intensity of emitted light upon electrochemical stimulation. Each sample’s response value was corrected for background response detected in 2R-injected mouse samples prior to interpolation of poly(GP) levels using the standard curve.

### Immunohistochemistry and digital analysis

Paraffin-embedded mouse brain tissues were cut on a sagittal plane at a thickness of 5 μm, deparaffinized with xylene, and rehydrated in a series of ethanol washes. For slides stained with mouse anti-NeuN (ABN78; Millipore) or rabbit anti-pTdp-43 (TIP-PTD-P01; pSer409/410; Cosmo Bio USA, Carlsbad, CA), antigen retrieval was performed by steaming slides for 30 min with distilled water before blocking in 0.03% hydrogen peroxide. Immunostaining of sections was done using a Dako Autostainer and Envision + HRP system (Dako, Carpintaria, CA) per the manufacturer’s instructions. For slides stained with rabbit anti-pTdp-43 (pSer409/410; gift from Dr. Leonard Petrucelli), antigen retrieval was performed by steaming slides for 30 min in sodium citrate buffer (10 mM sodium citrate, pH 6.0 with 0.05% Tween-20) and immunostaining was performed using the VectaStain Elite ABC HRP kit (Vector Laboratories, Burlingame, CA) per the manufacturer’s instructions. All slides were counterstained with hematoxylin, washed in a series of alcohols, and dehydrated in xylene. Glass coverslips were mounted using Cytoseal XYL (Thermo Scientific) and were left to set at room temperature for 48 h before scanning with an Aperio ScanScope AT2 Slide Scanner (Leica Biosystems, Buffalo Grove, IL). ImageScope software (v12.1.0.5029; Leica) was used to annotate the cerebral cortex and motor cortex of NeuN- and pTdp-43-stained slides. A custom-designed algorithm was applied to detect the number of pTdp-43-positive nuclei per area (mm^2^) [12] when labeled with the Cosmo antibody. The total number of pTdp-43-positive cells were counted manually for slides stained with the pTdp-43 antibody provided by Dr. Petrucelli.

### RNA fluorescence in situ hybridization (FISH)

RNA FISH was performed in fixed mouse brain tissue as done previously [12, 28]. Briefly, paraffin embedded brain sections were deparaffinized in xylene and rehydrated in a series of ethanol solutions. Sections were permeabilized with ice-cold 2% acetone in PBS prepared in DEPC-treated water for 5 min. Sections were then washed twice with DEPC-treated water and dehydrated in a series of ethanol solutions before incubating 30 min at 66 °C in pre-hybridization buffer [50% formamide/2X SSC (MIDSCI, Valley Park, MO), 10% dextran sulfate (Millipore), 2× saline-sodium citrate buffer, 50 mM sodium phosphate buffer, pH 7.0]. A fluorescently labeled locked nucleic acid (LNA) probe (5’-TYE563/CCCCGGCCCCGGCCCC-3′, Exiqon, Inc.; batch number 612968) was diluted to 40 nM in in hybridization buffer (10% dextran sulfate, 50% formamide, 20 ng/μl BSA, 25 mM tRNA, 25 nM EDTA, 2X SSC, 25 mM sodium phosphate buffer) and denatured at 80 °C for 5 min before hybridizing to the tissue for 24 h at 66 °C in a dark, humid chamber. Sections were subsequently washed once with 2X SSC (0.1% Tween-20) at room temperature for 5 min and washed twice with pre-warmed 0.2X SSC at 55 °C for 10 min in the dark. Slides were mounted with Vectashield mounting media containing DAPI (Vector Laboratories). Cortical images of RNA foci were obtained using a Zeiss Axio Imager Z1 fluorescent microscope (Zeiss, Oberkochen, Germany) under 63× magnification and the number of cells containing RNA foci were quantified.

### Statistical analyses

For experiments in which only two groups were compared, significance was measured using a Student’s t-test. For analyses involving more than two groups, GraphPad Prism 5.04 (GraphPad Software) was utilized to perform a one-way ANOVA or two-way ANOVA followed by the Fisher’s LSD post hoc test.

## Results

### Generation and validation of *Tmem106b* knockout mice

**Fig. 1:**
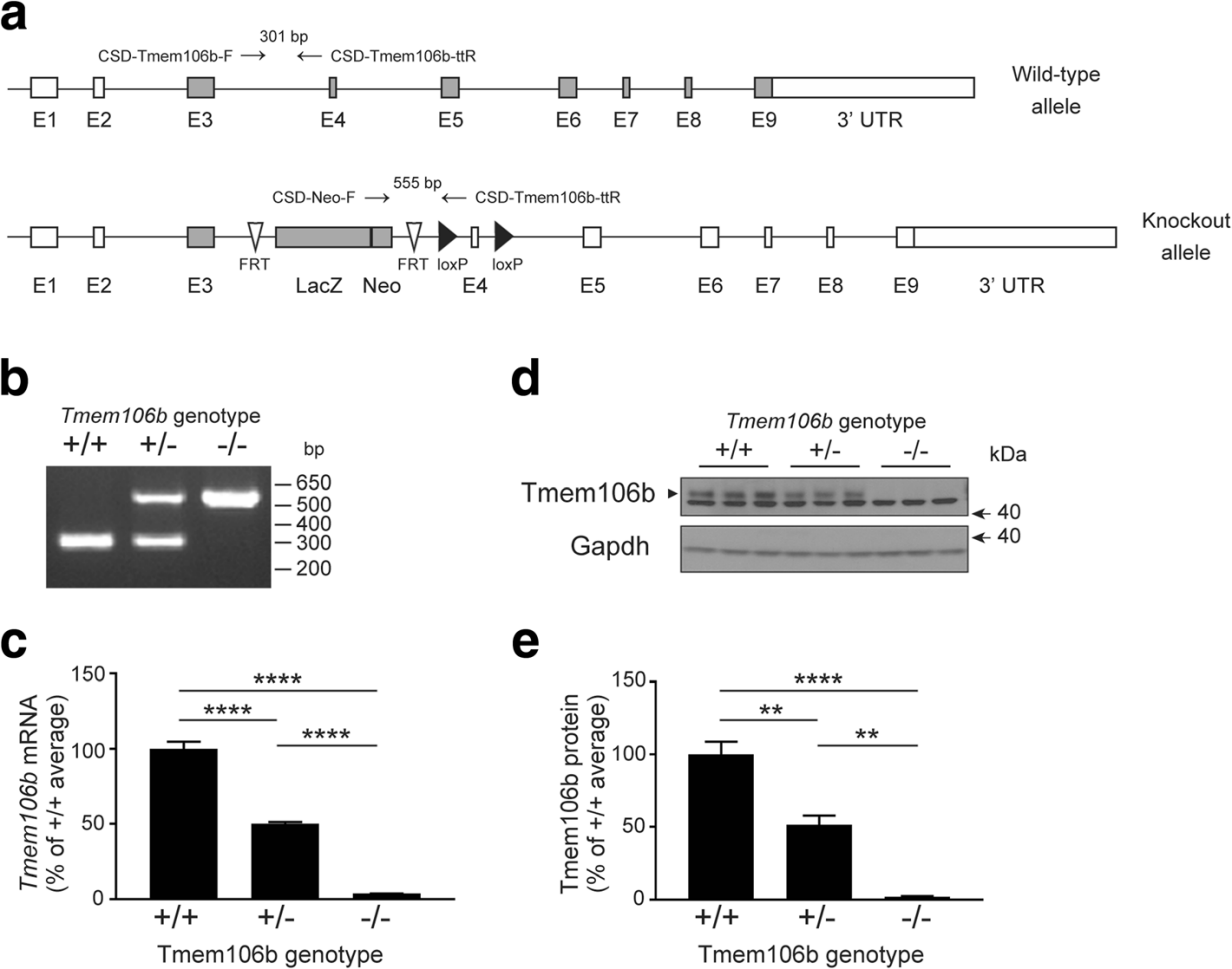
Generation of mice with targeted *Tmem106b* gene disruption. **a** Genomic structure of the wild-type mouse *Tmem106b* allele and the gene trap vector used to target the mouse *Tmem106b* gene. Rectangular boxes represent exons that are shaded in gray or white to denote coding and non-coding exons, respectively. White arrowheads signify Flp recombination target (FRT) sites and black arrowheads represent loci of crossover in P1 (LoxP) sites. Genotyping primer binding sites are labeled and denoted with black arrows above each genomic structure. **b** PCR analysis of DNA obtained from the hair follicles of *Tmem106b* wild-type (+/+), heterozygous (+/−), or knockout (−/−) mice using the primers depicted in panel a. **c** Quantitative PCR analysis measuring *Tmem106b* mRNA levels in *Tmem106b* +/+, +/−, and −/− mouse brain (*n* = 4 per genotype) at 3 months of age. The graph represents the mean ± S.E.M.; ****p < 0.0001 by one-way ANOVA followed by a Fisher’s LSD post-hoc test. **d** Western blot depicting Tmem106b protein levels (black arrowhead) in 3-month-old *Tmem106b* +/+, +/−, and −/− mouse brain tissue. Gapdh was used as a loading control. **e** Quantification of Tmem106b protein levels in Tmem106b +/+, +/-, and -/- mouse brain at 3 months of age (*n* =3 per genotype)

*Tmem106b* knockout alleles were generated by insertion of a lacZ gene trap in the intronic region between the first two coding exons, exons 3 and 4. The inserted gene is transcribed by the endogenous *Tmem106b* promoter along with the upstream exons and leads to a premature termination of transcription (Fig. 1a). Inheritance of this targeted gene disruption was confirmed by PCR amplification of the genomic DNA isolated from *Tmem106b* +/+, +/−, and −/− mice (Fig. 1b). qPCR analysis in 3, 8 and 15 months old mice further confirmed the loss of *Tmem106b* mRNA transcripts with mice heterozygous for the knockout allele showing approximately 50% loss as compared to age matched wild-type mice and a near complete loss of *Tmem106b* mRNA in *Tmem106b* −/− mice (Fig. 1c, Additional file 1**:** Figure S1). Loss of *Tmem106b* transcripts did not alter the expression of other Tmem106 family members, *Tmem106a* and *Tmem106c* (Additional file 1: Figure S2). Western blotting of wild-type mouse brain lysates with a Tmem106b antibody (Bethyl Laboratories) revealed a robust Tmem106b-immunoreactive band at the predicted 43 kDa molecular weight. This band’s intensity was reduced approximately 50% in samples from *Tmem106b* +/− mice and was undetectable in *Tmem106b* −/− brain tissue (Fig. 1d, e). Of note, using an in-house developed Tmem106b antibody against the intracellular domain of TMEM106B (residues 1–96) [7], small molecular weight bands could also be detected upon long exposure in *Tmem106b* +/− and *Tmem106b* −/− mice. These additional bands likely correspond to Tmem106b-lacZ truncated fragments which are expected in mouse models created by the lacZ gene trap approach [9] (Additional file 1: Figure S3).

### Tmem106b loss does not reverse abnormal behaviors in mice expressing (GGGGCC)_66_ repeat

**Fig. 2:**
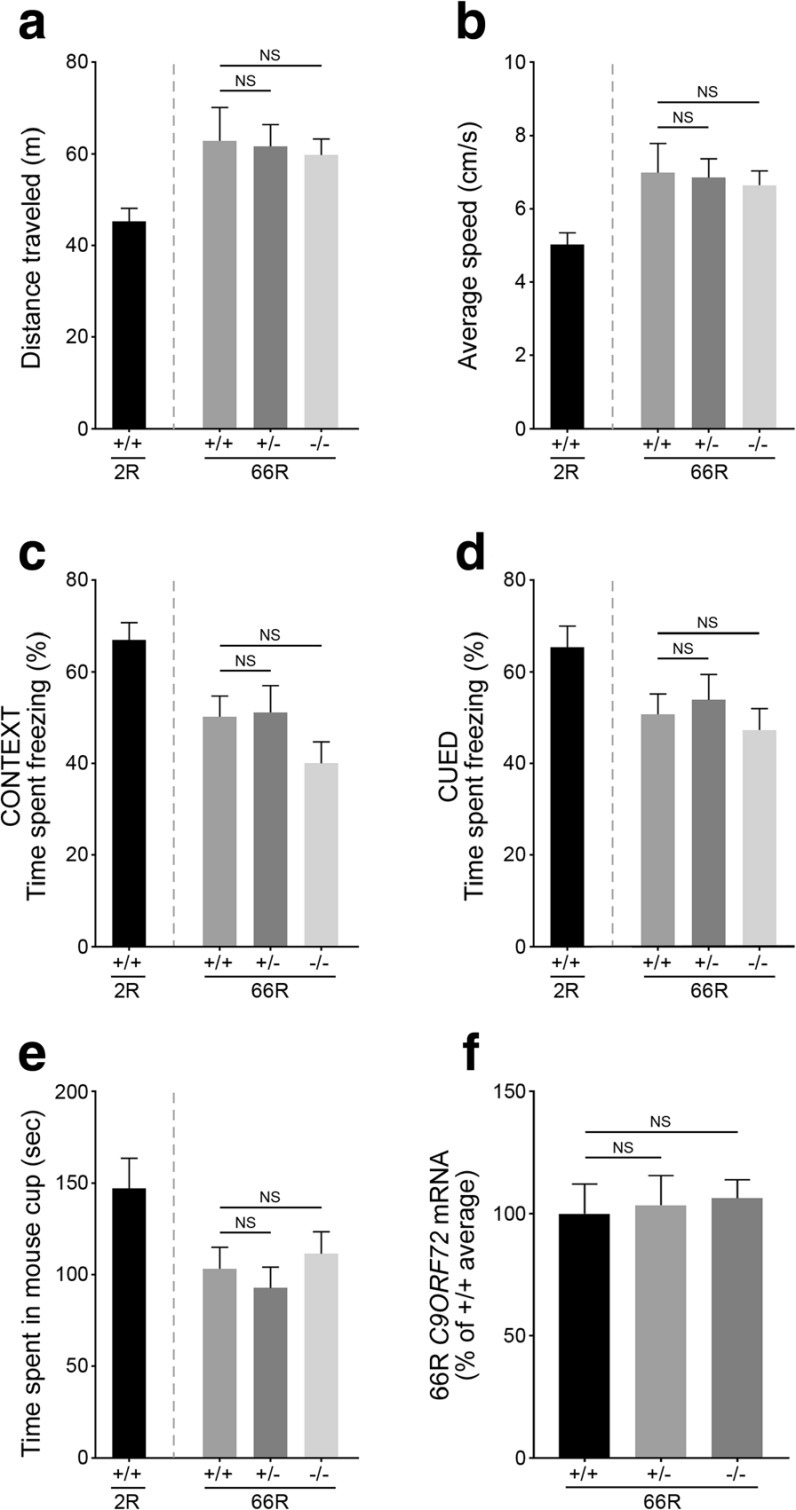
(GGGGCC)_66_ repeat-induced behavioral deficits are unchanged by Tmem106b reduction. **a** Quantification of the average distance traveled obtained during the open field assay for 66R-injected *Tmem106b* +/+, +/−, −/− mice at 12 months of age. Wild-type mice of the same age that had been injected with 2R *C9ORF72* were used as a control. **b** Quantification of the average speed traveled during the open field assay for mice as described in (**a**). **c-d** Quantification of the time each mouse spent freezing during the contextual (**c**) and cued (**d**) fear conditioning tests. **e** Quantification of the time each mouse spent exploring the mouse-containing cup during the social interaction test (**f** qPCR quantification measuring the amount of 66R mRNA obtained from the brains of 12-month-old 66R-injected *Tmem106b* +/+, +/−, and −/− mice. Graphs represent the mean ± S.E.M. Data was analyzed by one-way ANOVA followed by Fisher’s LSD post-hoc, and results are shown for 66R-injected *Tmem106b* +/− and −/− mice as compared to 66R-injected wild-type mice (*n* ≥ 12 for all groups). NS, not significant

To determine whether reduced Tmem106b levels might be protective against FTD-like behavioral phenotypes in the AAV-(GGGGCC)_66_ mouse model, the newborn *Tmem106b* +/+, +/−, and −/− mice were injected with either AAV- 66R or AAV-2R (control) and aged to 12 months of age. 66R virus injection did not affect the overall activity (data not shown) or body weight of the mice; except for a reduced body weight in female *Tmem106b* +/+ mice injected with 66R as compared to 2R which could be contributed to the small number of mice in this sub-group (Additional file 1: Figure S4). In line with the original study describing this model [12], injection of the 66R virus into wild-type mice induced significant behavioral deficits, including anxiety and reduced sociability as compared to 2R-injected wild-type mice (*p*=0.019 by Student’s t-test for all wild-type 2R vs. 66R analyses; Fig. 2a-e). However, neither partial nor complete reduction of Tmem106b altered the anxiety phenotype observed in 66R-injected wild-type mice as determined by the open field assay (Fig. 2a, b) or fear conditioning tests (Fig. 2c, d). Also, the reduced mouse sociability observed in 66R-injected mice could not be rescued in mice with partial or complete loss of Tmem106b (Fig. 2e). Since the amount of 66R viral expression could affect the presence and/or severity of these phenotypes, we performed qPCR analysis on all 66R-injected mouse brains, which showed no significant difference in 66R mRNA expression among *Tmem106b* +/+, +/−, and −/− mice (Fig. 2f).

### (GGGGCC)_66_ repeat-induced neuropathology is not rescued by lowering Tmem106b expression

**Fig. 3:**
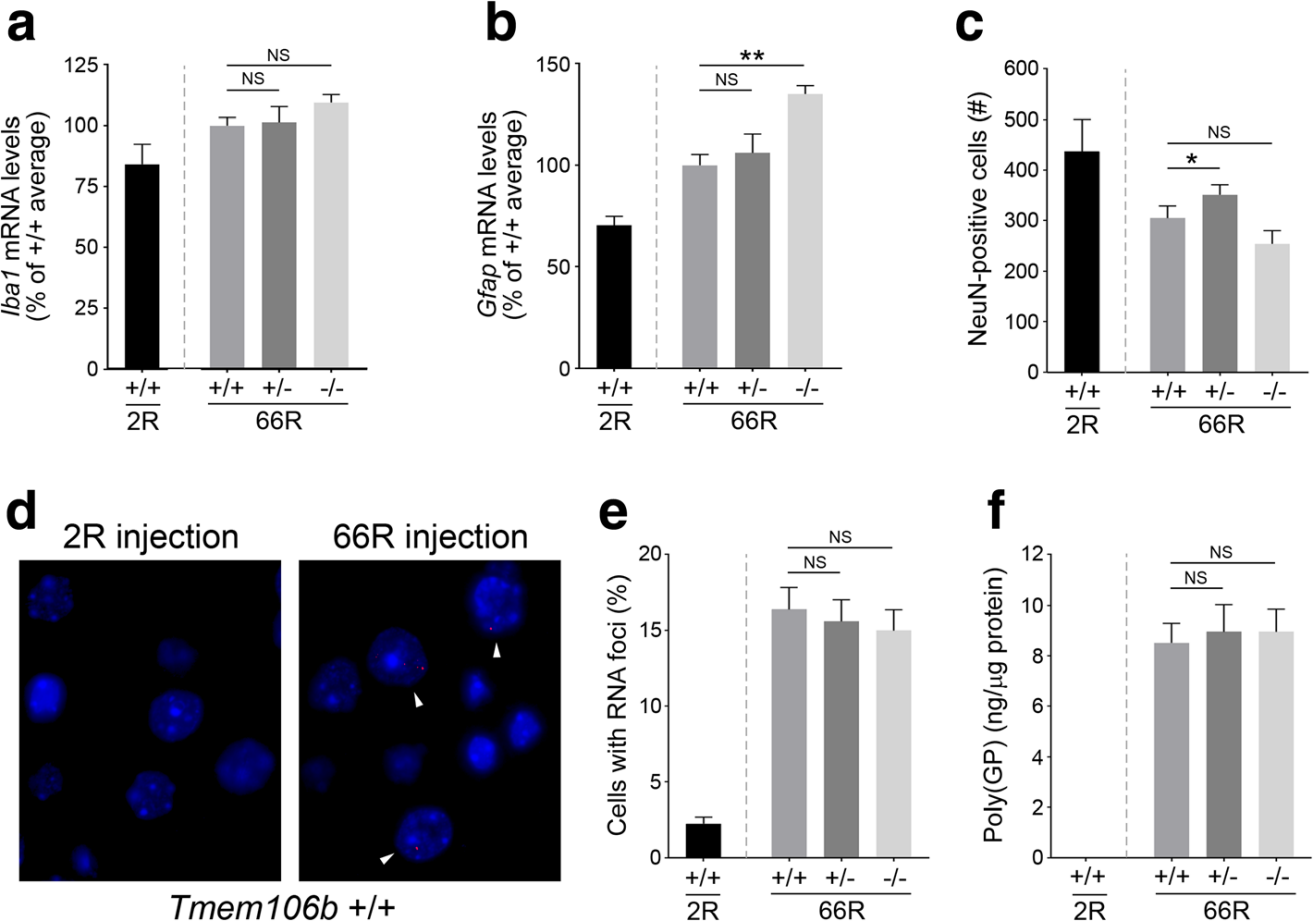
Assessment of (GGGGCC)_66_-mediated neuropathology in response to changes in Tmem106b levels (**a**-**b**) Quantification of *Iba1* (**a**) or *Gfap* (**b**) mRNA levels in the cortex of 2R-injected mice, or in 66R-injected *Tmem106b* +/+, +/−, or −/− mice. **c** Quantitative analysis of the number of cells immunoreactive for NeuN in the cortex of 2R-injected mice, or in 66R-injected *Tmem106b* +/+, +/−, or −/− mice. **d** Image depicting the presence of RNA foci (arrowheads) in the nuclei of cortical cells in wild-type 66R- versus 2R-injected mice. **e** Quantification of the number of cells with RNA foci in the cortex of wild-type 2R-injected mice, or in 66R-injected *Tmem106b* +/+, +/−, or −/− mice. **f** Quantitative assessment of the presence of poly(GP) peptides detected in the brains of mice injected as described in panel e. Graphs represent the mean ± S.E.M. Data was analyzed by one-way ANOVA followed by Fisher’s LSD post-hoc, and results are shown for 66R-injected *Tmem106b* +/− and −/− mice as compared to 66R-injected wild-type mice (*n* = 12 per group). NS, not significant.; *p < 0.05; **p < 0.001

We next determined whether lowering Tmem106b expression was able to ameliorate key hallmarks of neurodegeneration, such as neuroinflammation and neuronal loss, previously reported in the AAV-66R mouse model [12]. At 12 months of age, wild-type mice injected with 66R virus showed significantly increased levels of *Iba1* and *Gfap* mRNA transcripts as compared to 2R-injected animals, indicating pronounced neuroinflammation (*p* = 0.024 by Student’s t-test for both analyses; Fig. 3a, b). However, neither partial nor complete reduction of Tmem106b levels was able to rescue these changes; in fact, 66R-injected *Tmem106b* −/− mice had significantly higher *Gfap* mRNA levels than 66R-injected wild-type mice (Fig. 3b). Follow-up analysis in uninjected *Tmem106b* +/+, +/−, and −/− mice further showed that full loss of Tmem106b (in the absence of repeat overexpression), is sufficient to induce astrogliosis as determined by a significant increase in *Gfap* mRNA levels in *Tmem106b* −/− as compared to *Tmem106* +/+ mice (Additional file 1: Figure S5). *Gfap* mRNA levels in *Tmem106b* +/− mice were not significantly different from *Tmem106b* +/+ mice.

We next assessed the effect of Tmem106b reduction on 66R-induced neuronal loss. As expected, 66R-injected *Tmem106b* +/+ mice portrayed a significantly reduced number of cells immunoreactive for the neuronal marker, NeuN, as compared to 2R-injected mice (*p* = 0.016 by Student’s t-test; Fig. 3c). Complete loss of Tmem106b did not modify this phenotype; however, partial reduction of Tmem106b significantly lessened the extent of neuronal loss associated with 66R injection. In fact, 66R-injected *Tmem106b* +/− cortical NeuN counts were not significantly different from that of 2R-injected animals (*p* = 0.0512 by Student’s t-test; Fig. 3c**,** Additional file 1: Figure S6). This was not due to the presence of more NeuN-positive cells present in *Tmem106b* heterozygous mice given that uninjected *Tmem106b* +/+, +/−, and −/− mice have comparable NeuN counts (Additional file 1: Figure S5).

We next studied two unique pathologies induced by overexpression of the 66R repeat: RNA foci and dipeptide repeat proteins. Parallel to what was previously reported, RNA foci and poly-glycine/proline dipeptides [poly(GP)] were detected at significant levels in 66R-injected wild-type mice and not 2R-injected mice (Fig. 3d-f). We quantified the percentage of cells containing one or more RNA foci in the cortex of 66R-injected *Tmem106b* +/+, +/−, and −/− mice. As depicted in Fig. 3e, partial or complete reduction of Tmem106b levels did not significantly change the number of RNA foci-containing cells. Similarly, poly(GP) levels in 66R-injected *Tmem106b* +/− and −/− mice were similar to that of 66R-injected wild-type mice (Fig. 3f).

**Fig. 4:**
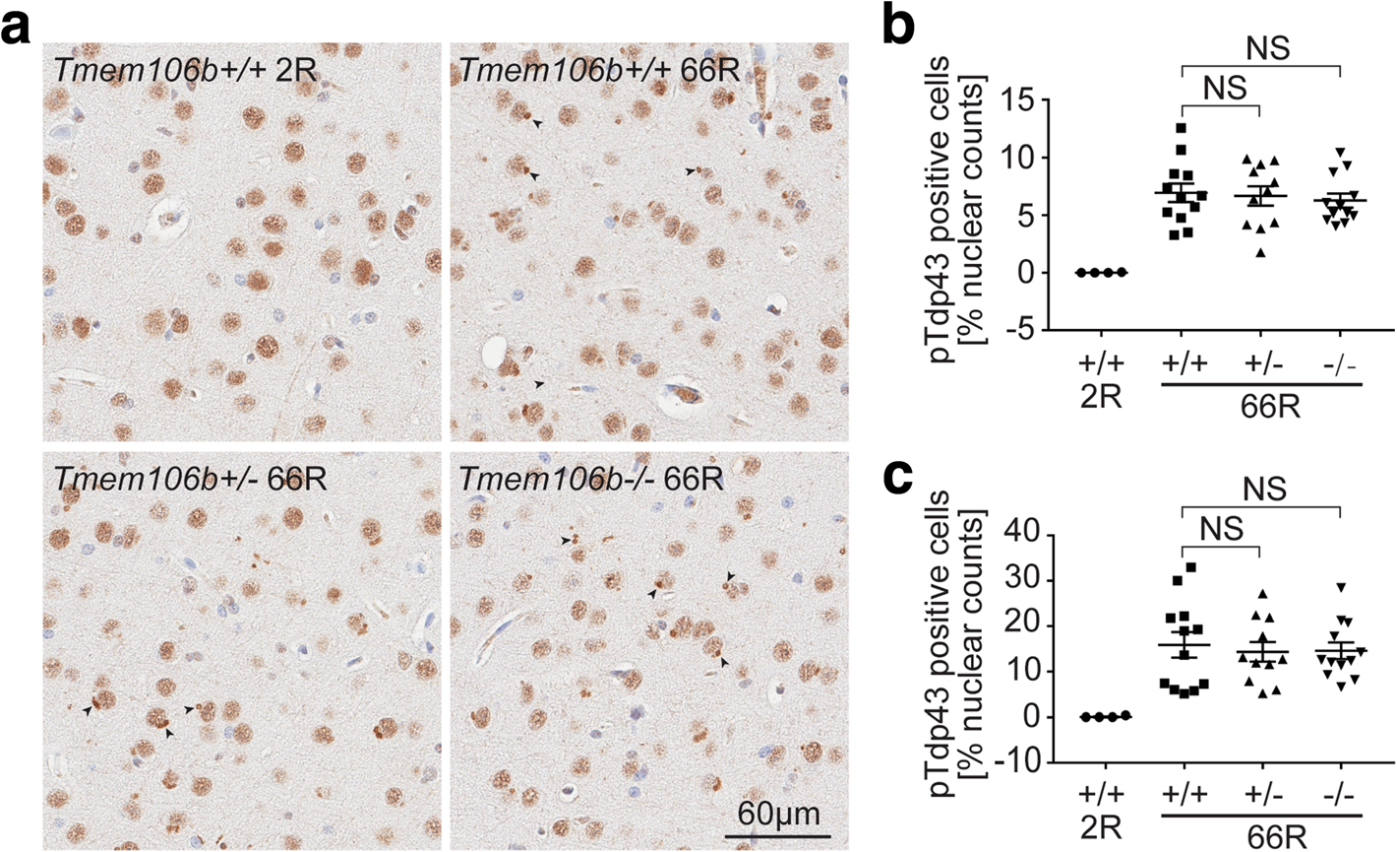
Tmem106b levels do not affect (GGGGCC)_66_ repeat induced pTdp-43 inclusion body formation. **a** Representative images of pTdp-43 (pS409/410 from Cosmo Bio) staining of motor cortex region of the mouse brains from indicated *Tmem106b* genotypes 12 months after 2R and 66R AAV injection. Arrow heads indicate pTdp-43-positive inclusion bodies. **b** and **c** Quantification of pTdp-43 inclusion body positive cells in different brain regions: cortex (**b**) or hippocampus (**c**) from *Tmem106b* +/+, +/−, and −/− mice as compared to WT 2R injected mice (*n* = 4). Graphs represent the mean ± S.E.M. Data was analyzed by one-way ANOVA followed by Fisher’s LSD post-hoc, and results are shown for 66R-injected *Tmem106b* +/− and −/− mice as compared to 66R-injected wild-type mice (*n* = 12 per group). NS, not significant

Finally, we examined the effect of Tmem106b reduction on the formation of intracellular inclusions of phosphorylated Tdp-43 (pTdp-43), a key pathological feature observed both in patients and in mice injected with 66R [12, 17, 45]. We detected intense pTdp-43-positive inclusions in 66R- but not 2R-injected *Tmem106b +/+* brains using two different antibodies that recognize Tdp-43 in its phosphorylated form (Fig. 4a, Additional file 1: Figure S7). However, neither partial nor complete loss of Tmem106b significantly changed the number of pTdp-43-positive cells in the cortex or hippocampus (Fig. 4b; Additional file 1: Figure S7).

### *C9ORF72* loss increases TMEM106B levels unlike (GGGGCC)_66_ overexpression

**Fig. 5:**
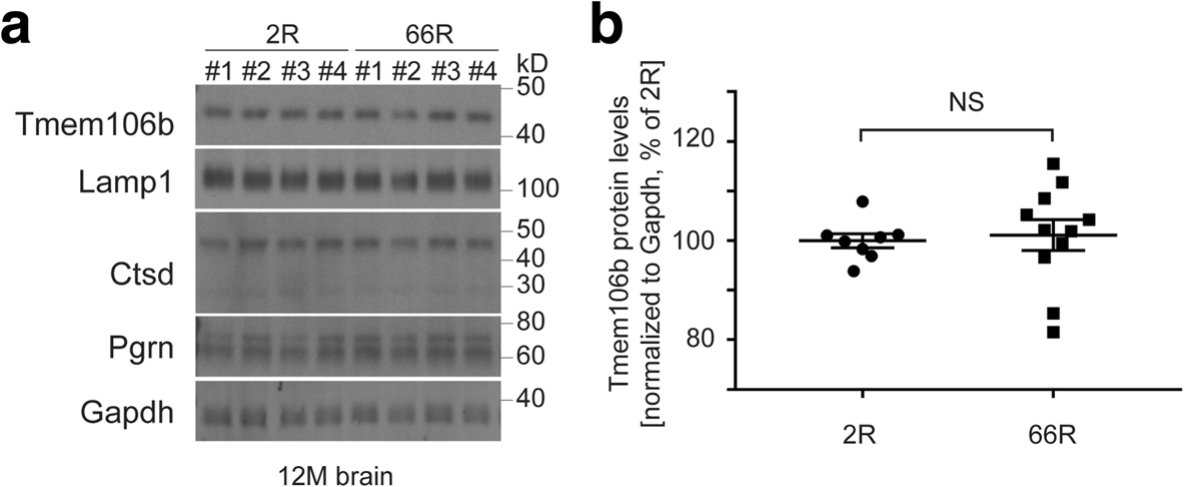
(GGGGCC)_66_ repeat expansion overexpression does not alter Tmem106b protein levels in mouse brain. (**a**) Western blot of brain tissue obtained from 2R- or 66R-injected wild-type mice using antibodies against various lysosomal proteins. Gapdh was used as a loading control. (**b**) Quantification of Tmem106b protein levels by Western blot as depicted in panel a. The graph represents the mean ± S.E.M. by Student’s *t*-test (*n* = 8 for 2R; n = 12 for 66R ); NS, not significant

**Fig. 6:**
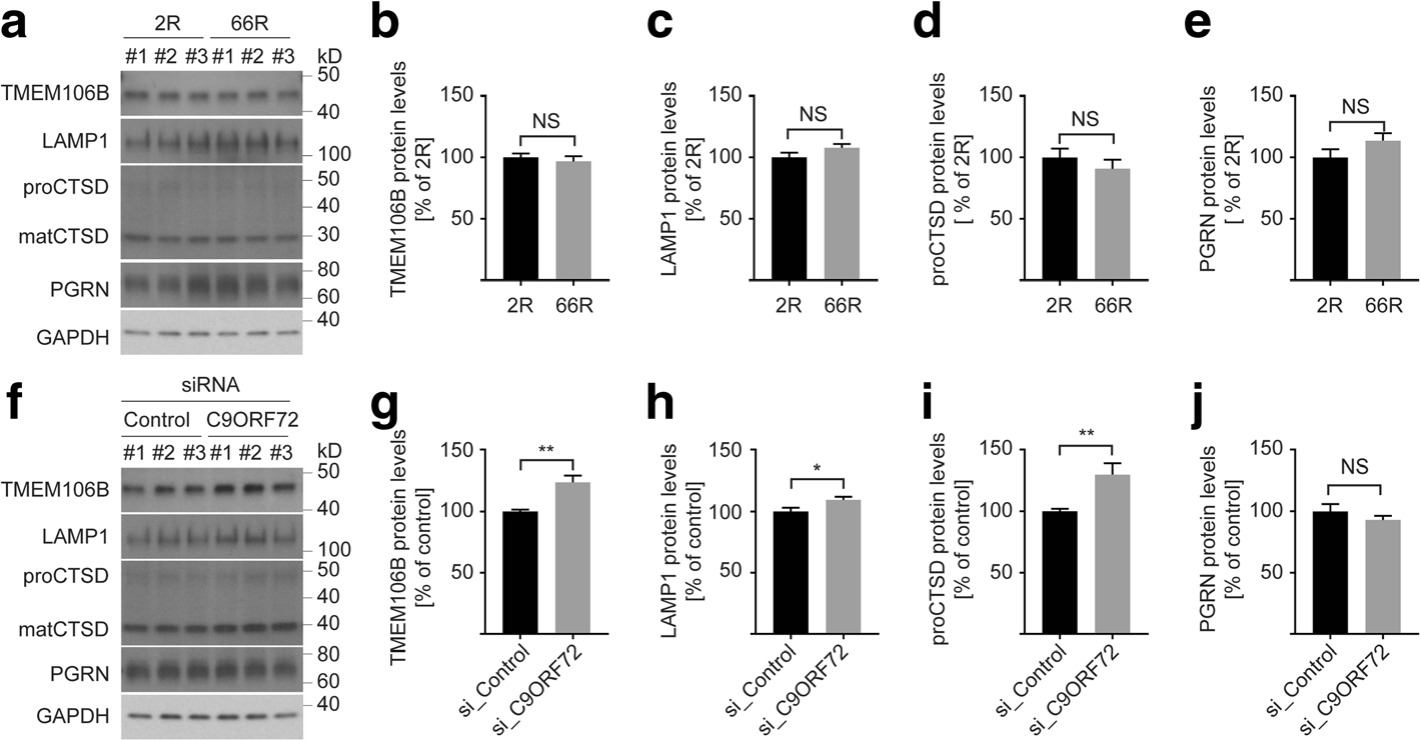
The effect of (GGGGCC)_66_ overexpression or *C9ORF72* knockdown on TMEM106B protein levels in HeLa cells. **a** Western blot of HeLa cells transfected with either 2R or 66R pAAV. **b**-**e** Protein quantification of TMEM106B (**b**), LAMP1 (**c**), CTSD (pro-form, mature form has similar results as the pro-form) (**d**), and PGRN (**e**) in cells transfected as described in panel (**a**). **f** Western blot of HeLa cells transfected with either control siRNA or siRNA against *C9ORF72* (**g-j**), Protein quantification of TMEM106B (**g**), LAMP1 (**h**), CTSD (**i**), and PGRN (**j**) in cells transfected as described in panel f. GAPDH was used as a loading control. Graphs represent the mean ± S.E.M. by Student’s t-test (*n* = 6 for all groups). NS, not significant; *p < 0.05, **p < 0.01

To further study the connection between TMEM106B and *C9ORF72*-related disease mechanisms, we determined Tmem106b protein levels in wild-type mice that had been injected with 66R or 2R virus. Tmem106b levels in 66R injected brains were almost identical to that of 2R control brains (Fig. 5a, b). The 66R-injected mice also had normal levels of other lysosomal proteins, such as Lamp1, cathepsin D (both pro- and mature forms), and progranulin (Fig. 5a, quantifications not shown). Furthermore, whereas human patients with *C9ORF72*-related FTD have reduced *C9ORF72* levels in addition to the GGGGCC-repeat associated toxicities, endogenous *C9ORF72* expression levels were not changed in our 66R overexpression model (Additional file 1: Figure S8). This prompted us to compare the effects of 66R overexpression and *C9ORF72* loss on Tmem106b levels in cell culture (techniques validated in Additional file 1: Figure S9). Consistent with the in vivo data, overexpression of 66R in both HeLa and U251 cells failed to change the expression of various lysosomal proteins, including TMEM106B (Fig. 6a-e and Additional file 1: Figure S10). However, knockdown of *C9ORF72* significantly increased protein levels of TMEM106B together with other lysosomal resident proteins (Fig. 6f-j and Additional file 1: Figure S10).

## Discussion

Genetic variants in *TMEM106B* associated with reduced TMEM106B expression have been shown to significantly protect individuals with either *GRN* mutations or *C9ORF72* repeat expansions from the development of FTD symptoms [13, 19, 21, 30, 37, 58–60]. Moreover, depletion of Tmem106b was recently shown to rescue several disease-relevant phenotypes observed in *Grn* −/− mice [26]. This study is the first to determine the impact of Tmem106b reduction on *C9ORF72*-related disease. Contrary to the relative success in the *Grn* −/− mouse model, we demonstrate that neither partial nor complete loss of Tmem106b is sufficient to rescue the behavioral changes or neuropathological phenotypes that manifest in an AAV-based mouse model of *C9ORF72*-associated (GGGGCC)_n_ expansions. The (GGGGCC)_66_-AAV model was chosen as the model system in this study due to the demonstration of various phenotypes associated with *C9ORF72* repeat expansions as early as 6 months of age [12]. At the time this study commenced, only two other characterized *C9ORF72*-repeat expansion mouse models had been generated. Both of these models were created using a bacterial artificial chromosome (BAC) for the expression of the complete [39] or partial [41] *C9ORF72* coding region with incorporated (GGGGCC) repeats of various lengths. These models successfully demonstrated pathologies directly related to the repeat expansion as early as 4–6 months of age, including RNA foci and the generation of dipeptide repeat proteins. However, in contrast to the (GGGGCC)_66_-AAV model, many of the key features of *C9ORF72*-mediated FTD, especially behavioral deficits, neuroinflammation, neuronal loss, and pTdp-43 pathology, were not observed even in aged mice in these models [39, 41]. Since TDP-43 pathology is a common denominator of the human disease populations associated with *TMEM106B* (FTLD-TDP, *GRN*-carriers, *C9ORF72*-carriers and AD patients with TDP-43 pathology [38]) the selection of a mouse model with pTdp-43 pathology was considered essential. Importantly however, neither the BAC mice nor our (GGGGCC)_66_-AAV mice recapitulate the loss of *C9ORF72* expression consistently observed in human *C9ORF72* expansion carriers.

Neuronal loss and neuroinflammation are common features among neurodegenerative disorders. Protective *TMEM106B* variants were found to associate with increased neuronal gene expression, reduced expression of genes involved in inflammation, and better cognitive performance in healthy aged individuals [46]. However, we did not see a significant improvement in measures of neuroinflammation or behavioral deficits induced by the overexpression of the (GGGGCC)_66_ repeat when Tmem106b levels were reduced. In fact, (GGGGCC)_66_-injected *Tmem106b* knockout mice showed more severe astrogliosis, evidenced by increased *Gfap* expression, as compared to *Tmem106b* wild-type or heterozygous mice injected with the expanded repeat. We showed that this was due to increased astrogliosis from Tmem106b loss alone, excluding the possibility that loss of Tmem106b renders mice more sensitive to a *C9ORF72* repeat-mediated inflammatory response. Our observation of astrogliosis resulting from loss of Tmem106b alone suggests that Tmem106b plays a unique and potentially necessary role in astrocytes. Tmem106b is a lysosomal resident protein, and loss of Tmem106b has been recently shown to cause lysosomal dysfunctions including lysosomal acidification and trafficking problems [26, 49]. Importantly, dysfunction of lysosomes in multiple diseases, for instance, lysosomal storage disorders has been tightly linked to astroglial activation [42]. Interestingly, activation of the Tmem106b paralog, Tmem106a, was shown to be immunostimulatory in mouse macrophages [15]. As such, our data suggests that TMEM106B might play a novel, reciprocal role in inflammatory modulation. In relation to the neuronal loss, we did observe that partial, but not complete loss of Tmem106b significantly lessened the extent of neuronal loss in the AAV-(GGGGCC)_66_ injected mice, suggesting that, if pursued, partial TMEM106B reduction may be a more viable avenue for future TMEM106B-related therapeutic approaches in FTD.

Tmem106b levels also had no effect on two other key pathological features observed in the (GGGGCC)_66_-AAV model: RNA foci and the generation of dipeptide repeat proteins. These data are in line with a previous report that did not find an association between *TMEM106B* variants and dipeptide repeat pathology in *C9ORF72* mutation carriers [16]. Given the strong genetic association of both *GRN* and *C9ORF72* carriers with *TMEM106B* variants, the lack of association in our Tmem106b model with pathological features that are unique to *C9ORF72*GGGGCC repeat expansion carriers may not be surprising. Nevertheless, both *C9ORF72* and *GRN* mutation carriers present with TDP-43 pathology at autopsy. Indeed, *TMEM106B* variants were first discovered as disease modifiers in an FTD cohort comprised of individuals with TDP-43 brain pathology regardless of underlying cause [59]. *TMEM106B* variants were additionally found to associate with the presence of TDP-43 pathology in other diseases, such as Alzheimer’s disease and hippocampal sclerosis [3, 35, 48]. In fact, the protective *TMEM106B* variants appeared to correspond with lessened TDP-43 aggregate burden in a preliminary study of eight *C9ORF72* mutation carriers [58]. Collectively, these findings suggest that *TMEM106B* protective variants may reduce one’s risk of developing TDP-43 proteinopathies; however, despite careful analysis with two independent antibodies, we found that reducing Tmem106b levels in (GGGGCC)_66_-injected mice did not lessen the development of phosphorylated pTdp-43 aggregates in the cortex or hippocampus at 12 months of age.

The inability to ameliorate neuropathological and behavioral deficits through the reduction of *Tmem106b* in the AAV-(GGGGCC)_66_ model requires a careful examination of the approaches employed in this study. First, we modelled the protective *TMEM106B* human haplotype by reducing *Tmem106b* by approximately 50 or 100%. This seems appropriate since multiple studies have observed lower *TMEM106B* mRNA levels or a faster TMEM106B protein degradation associated with the *TMEM106B* protective haplotype [20, 37, 59]. We cannot, however, exclude that the associated variants affect TMEM106B in an unknown fashion, for example as a result of the p.Thr185Ser variant (rs3173615) which alters the protein coding sequence of TMEM106B and is inherited as part of the protective haplotype [59]. It is also possible that the protective TMEM106B haplotype induces a more subtle decrease in TMEM106B than what we modeled in this study or that the N-terminal TMEM106B fragments, which we detected at low levels in knockout animals, may have retained some partial TMEM106B function. Second, we modelled the *C9ORF72*-associated repeat expansion through the overexpression of (GGGGCC)_66_ by AAV. This approach importantly results in pTdp-43-positive inclusions as early as 6 months of age [12]; however, this model may have been too aggressive to reverse the neurodegenerative, neuropathological, and behavioral phenotypes in these mice at 12 months of age. More importantly, our approach only recapitulated the RNA and protein toxic gain-of-function mechanisms associated with the repeat sequence and failed to model the reduction in *C9ORF72* transcripts which is now considered and integral part of *C9ORF72* disease pathogenesis [1, 17, 45, 56]. Indeed, endogenous C9orf72 levels were unchanged in our 66R mice at 12 months of age.

TMEM106B is a type II lysosomal membrane protein with currently unknown function. Increases in TMEM106B levels have been found to be cytotoxic and are associated with increases in lysosomal size and reduced lysosomal acidification, leading to the disruption of endolysosomal- and autophagic-lysosomal degradation [38]. Recent work undeniably links PGRN to lysosomal biology [24, 31, 64, 65] and, as such, it may not have been surprising that Tmem106b loss reversed some of the *Grn* knockout-mediated lysosomal enzyme dysregulation [26]. This prompts the question of whether Tmem106b reduction might only confer protection in disease models that portray abnormal lysosomal biology. While lysosomal dysfunction has been implicated in *C9ORF72*-related pathogenesis, much of the evidence to support this comes from studies investigating the function of the C9ORF72 protein itself and not the (GGGGCC)_n_ repeat expansions [2, 8, 27, 40, 52, 55]. The C9ORF72 protein sequence contains DENN-like domains, making it a part of the DENN protein superfamily which is known to be involved in regulating membrane trafficking and autophagy [32, 63]. Genetic and cell biology studies have shown that C9ORF72 interacts with other DENN domain-containing proteins linked to mTORC1 signaling, whose activity is closely tied to lysosomal function [2, 51, 55, 62]. Specifically, loss of C9ORF72, as seen in human patients carrying *C9ORF72* repeat expansions, causes impaired mTORC1 signaling and abnormal lysosome morphology indicative of dysfunction [2, 57]. Thus, it is conceivable that the effect of the protective *TMEM106B* haplotype in *C9ORF72* expansion carriers is to counteract lysosomal dysfunction that results from the loss of *C9ORF72* expression. In support of this hypothesis, we showed that reducing C9ORF72 levels in human cell lines significantly increased levels of various lysosomal proteins, including TMEM106B, which is thought to signify lysosomal dysfunction. These types of lysosomal changes were not observed by overexpression of (GGGGCC)_66_ in cell culture, nor were these changes observed in our AAV-(GGGGCC)_66_ mouse model.

## Conclusions

In summary, we show that reducing the levels of Tmem106b in a mouse model mimicking the toxic gain-of-functions associated with the *C9ORF72* (GGGGCC)_n_ repeat expansions is unable to ameliorate key pathological features seen in FTD patients, including pTdp-43 pathology. We further provide support for the growing body of evidence linking the loss of *C9ORF72* expression to the pathobiology of C9ORF72, in this case through the induction of lysosomal dysfunction. As such, it will be critically important that further examination of the effects of Tmem106b reduction on C9ORF72 pathobiology be studied in models that include reduction of C9ORF72.

## Additional file

Additional file 1: **Figures S1 through S10. Figure S1.** Transcript expression of *Tmem106b* in Tmem106b deficiency mice at different ages. **Figure S2.** Tmem106b reduction does not alter the expression of its family members. **Figure S3.** Tmem106b immunoreactivity in mice with *Tmem106b* gene interruption using an additional antibody. **Figure S4.** The body weight of 2R and 66R injected mouse. **Figure S5.** Tmem106b reduction alone induces astrogliosis. **Figure S6.** Heterozygous loss of Tmem106b partially rescues 66R injection-induced neuronal loss. **Figure S7.** pTdp-43 immunoreactivity in 2R and 66R injected mouse brain. **Figure S8.** Endogenous C9orf72 protein levels in 2R- and 66R-injected mouse brain. **Figure S9.** Validation of (GGGGCC)_66_ repeat overexpression and *C9ORF72* knockdown. **Figure S10.** The effect of (GGGGCC)_66_ overexpression or *C9ORF72* knockdown on TMEM106B protein levels in U251 cells. (DOCX 26231 kb)
