# Supplementary material for: Loss of Tmem106b is unable to ameliorate frontotemporal dementia-like phenotypes in an AAV mouse model of C9ORF72-repeat induced toxicity
Source: Acta Neuropathol Commun. 2018 May 31;6:42. doi: 10.1186/s40478-018-0545-x (PMC5984311; doi:10.1186/s40478-018-0545-x)
Supplement: Supplementary file 1 — Figures S1 through S10. Figure S1. Transcript expression of Tmem106b in Tmem106b deficiency mice at different ages. Figure S2. Tmem106b reduction does not alter the expression of its family members. Figure S3. Tmem106b immunoreactivity in mice with Tmem106b gene interruption using an additional antibody. Figure S4. The body weight of 2R and 66R injected mouse. Figure S5. Tmem106b reduction alone induces astrogliosis. Figure S6. Heterozygous loss of Tmem106b partially rescues 66R injection-induced neuronal loss. Figure S7. pTdp-43 immunoreactivity in 2R and 66R injected mouse brain. Figure S8. Endogenous C9orf72 protein levels in 2R- and 66R-injected mouse brain. Figure S9. Validation of (GGGGCC)66 repeat overexpression and C9ORF72 knockdown. Figure S10. The effect of (GGGGCC)66 overexpression or C9ORF72 knockdown on TMEM106B protein levels in U251 cells. (DOCX 26231 kb) [file 40478_2018_545_MOESM1_ESM.docx]

***Supplementary Information***

**Supplementary Figure 1.**


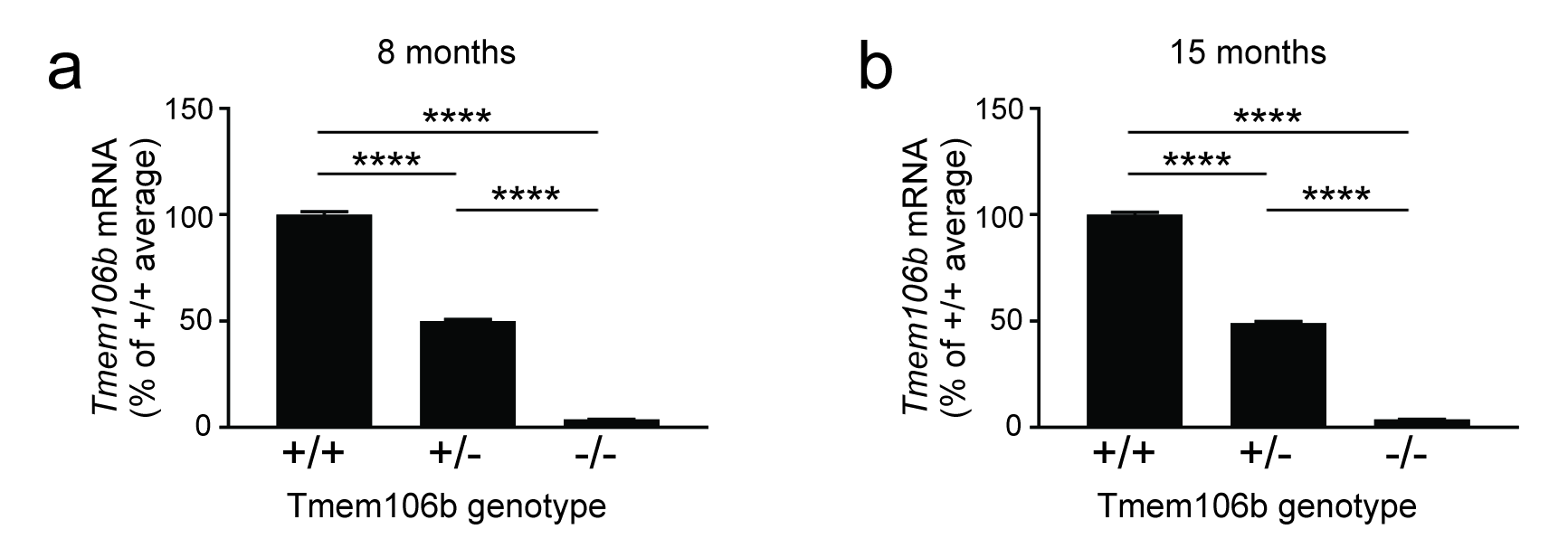


**Supplementary Figure 1. Transcript expression of *Tmem106b* in Tmem106b deficiency mice at different ages.** Quantitative PCR analysis measuring *Tmem106b* mRNA levels in *Tmem106b* +/+, +/-, and -/- mouse brain (n=4 per genotype) at 8 and 15 months of age. The graph represents the mean ± S.E.M.; ****p<0.0001 by one-way ANOVA followed by a Fisher’s LSD post-hoc test.

**Supplementary Figure 2**


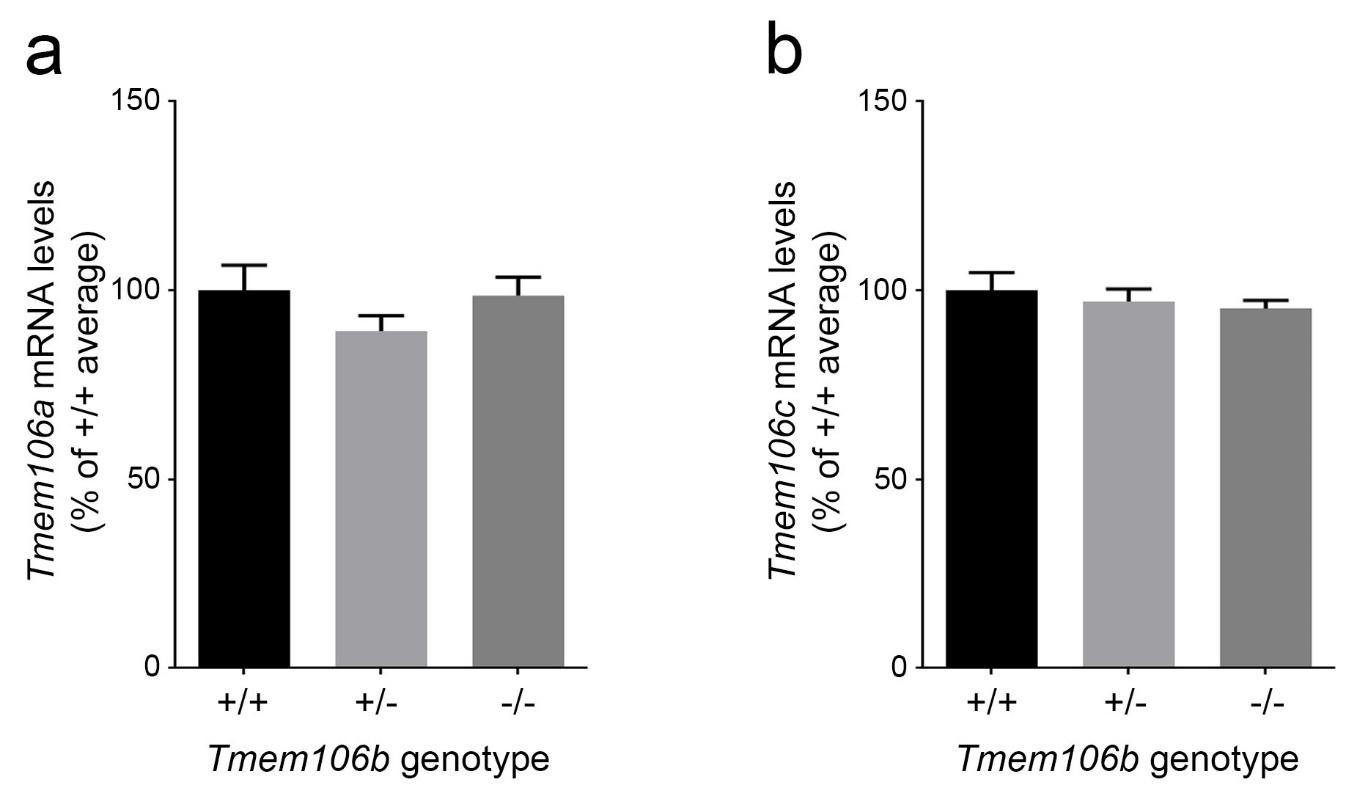


**Supplementary Figure 2. Tmem106b reduction does not alter the expression of its family members. (a-b)** Quantification of Tmem106a **(a)** and Tmem106c **(b)** mRNA levels in the brains of *Tmem106b* +/+, +/-, and -/- mice at 3 months of age (n=4 per genotype). Graphs represent the mean ± S.E.M. analyzed by one-way ANOVA followed by Fisher’s LSD post-hoc test.

**Supplementary Figure 3.**


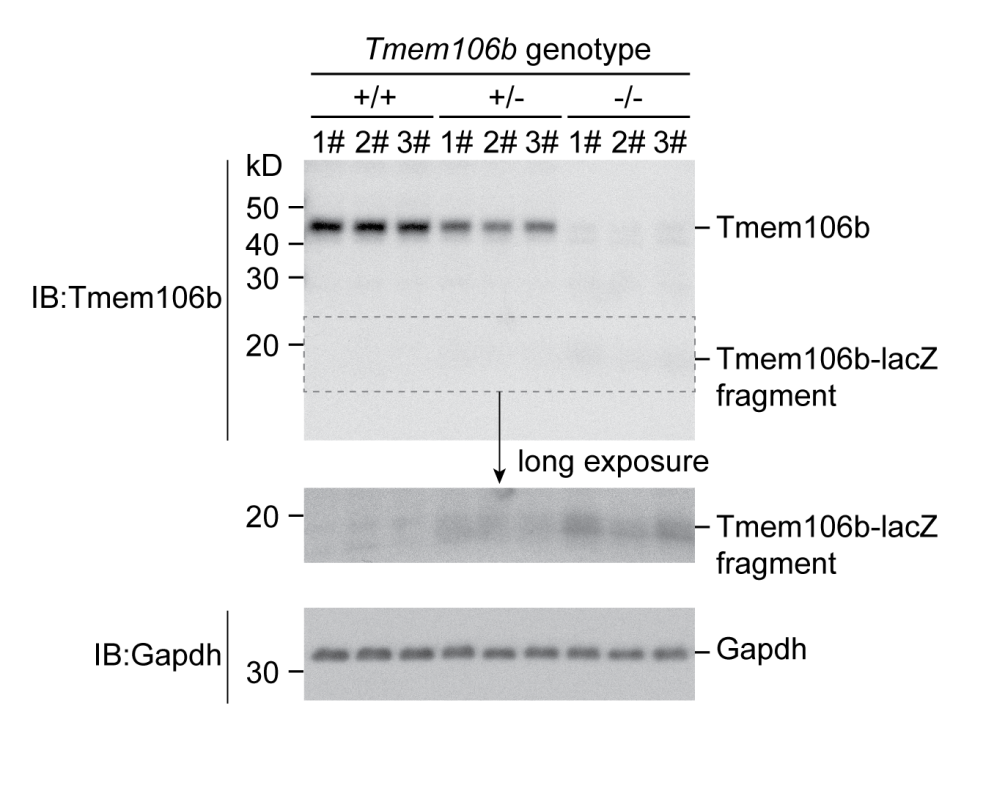


**Supplementary Figure 3. Tmem106b immunoreactivity in mice with *Tmem106b* gene interruption using an additional antibody.** Western blot depicting full-length Tmem106b and a small predicted N-terminal fragment in 3-month-old Tmem106b +/+, +/-, and -/- mouse brain tissue using a Tmem106b antibody generated by Dr. Fenghua Hu’s laboratory. Gapdh was used as a loading control.

**Supplementary Figure 4.**


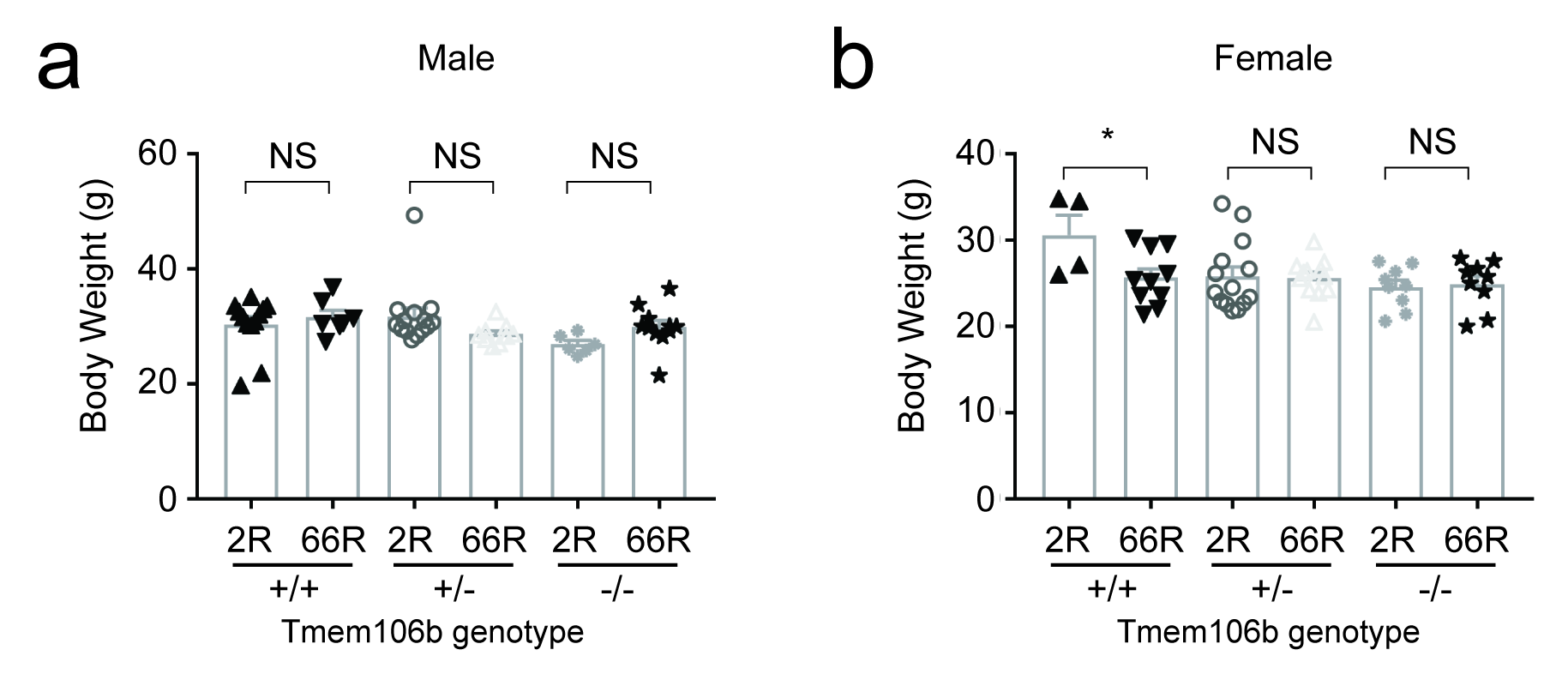
**Supplementary Figure 4. The body weight of 2R and 66R injected mouse.** The body weight of both male **(a)** and female **(b)** mice with different Tmem106b genotypes were measured 12 months after 2R or 66R injection (n=4 to 14 per group). Graphs represent the mean ± S.E.M. *p<0.05 by by Student’s t-test; NS, not significant.

**Supplementary Figure 5.**

**

**

**Supplementary Figure 5. Tmem106b reduction alone induces astrogliosis. (a)** Quantification of *Gfap* mRNA levels by qPCR in uninjected *Tmem106b* +/+, +/-, and -/- mice at 8 months of age (n=8 per group). **(b)** Quantification of NeuN-immunoreactive cells in the cortex of uninjected *Tmem106b* +/+, +/-, and -/- mice at 8-15 months of age (n≥7 per group). Graphs represent the mean ± S.E.M. ****p<0.0001 by one-way ANOVA followed by Fisher’s LSD post-hoc test.

**Supplementary Figure 6**

**
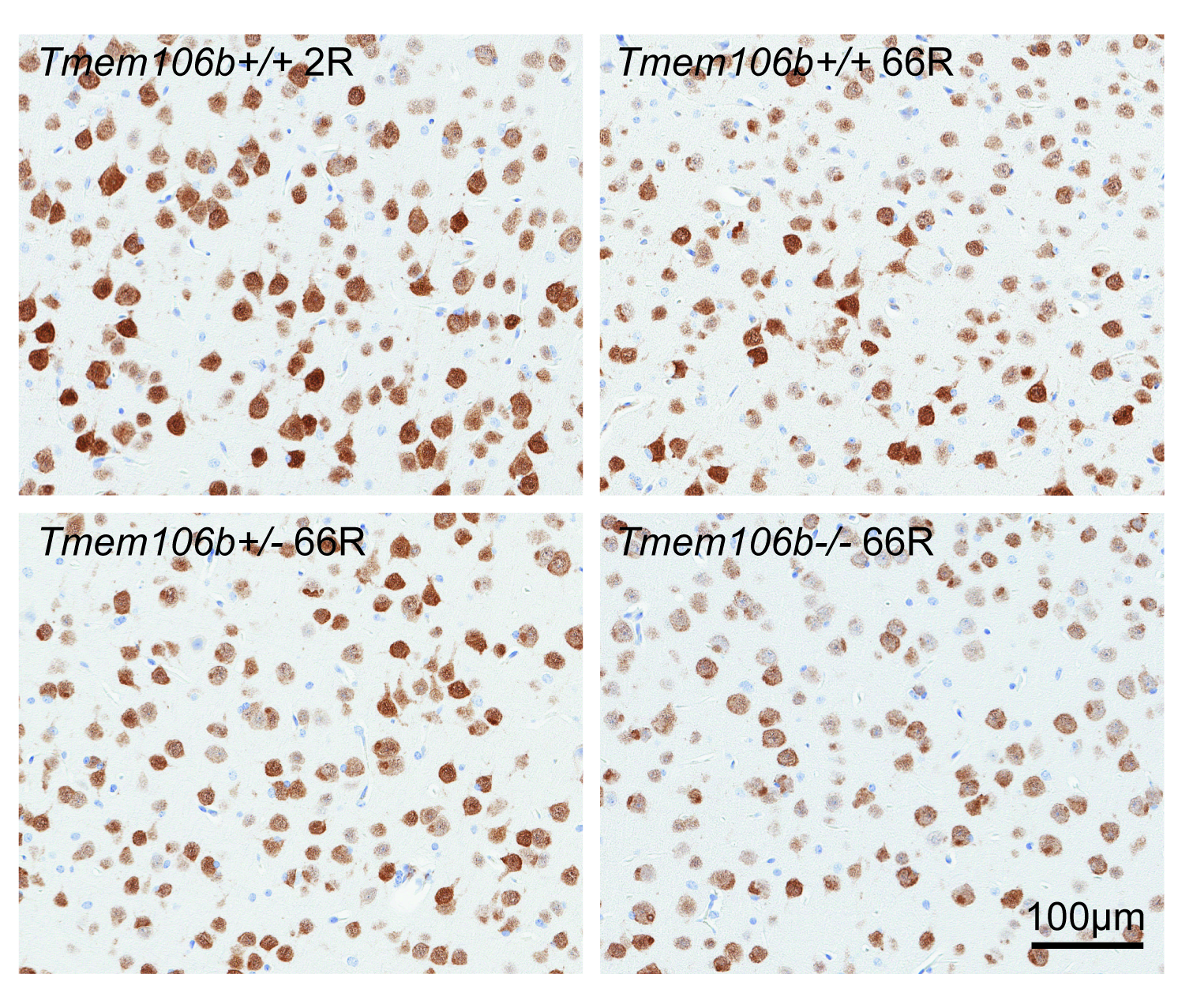
**

**Supplementary Figure 6. Heterozygous loss of Tmem106b partially rescues 66R injection-induced neuronal loss.** Representative images shows NeuN immunostaning (NeuN positive cells are in brown color of the motor cortex region (layers 3-4) of 2R or 6R injected *Tmem106b* +/+, +/-, and -/- mice at 12 months of age.

**Supplementary Figure 7**

**
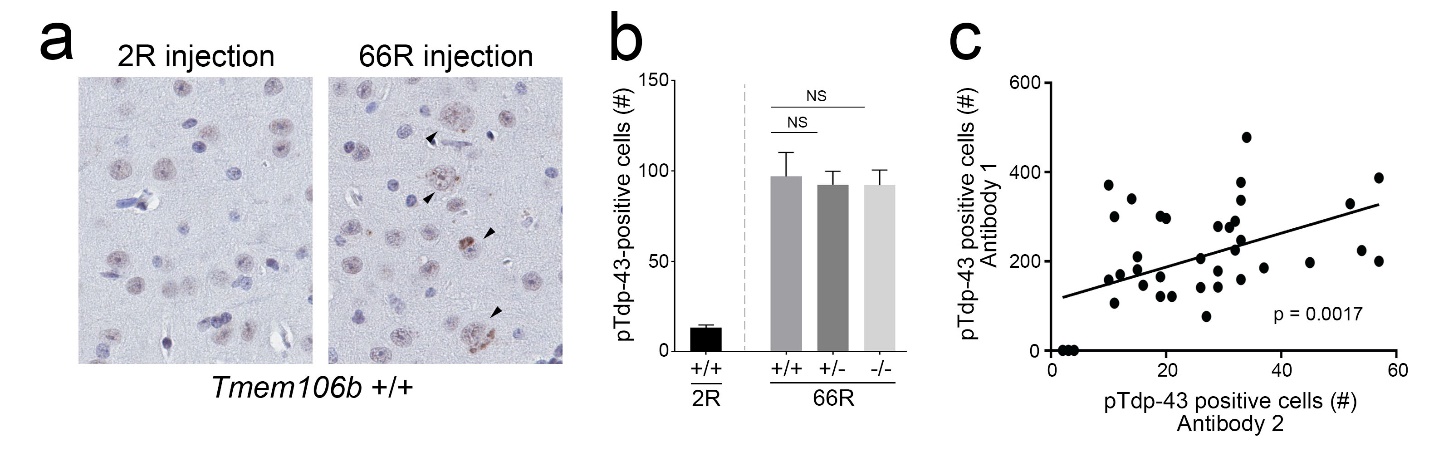
**

**Supplementary Figure 7. pTdp-43 immunoreactivity in 2R and 66R injected mouse brain. (a)** Representative images of pTdp-43 (pS409/410 antibody from Dr. Leonard Petrucelli) staining of the motor cortex region of mouse brains from the indicated *Tmem106b* genotypes 12 months after 2R and 66R AAV injection. Arrow heads indicate pTdp-43-positive cells. **(b)** Quantification of pTdp-43-positive cells in motor cortex from *Tmem106b* +/+, *Tmem106b* +/-, or *Tmem106b* -/- mice as compared to *Tmem106b* +/+ 2R injected mice. Graph represents the mean ± S.E.M. by one-way ANOVA followed by Fisher’s LSD post hoc test. NS, not significant. **(c)** Scatter plot illustrating the significant positive correlation (p=0.0017) between pTdp-43 positive cell counts obtained from immunostaining of with two pTdp-43 (pS409/401) antibodies, obtained from either Cosmo Bio (Antibody 1) or Dr. Leonard Petrucelli (Antibody 2).

**Supplementary Figure 8**


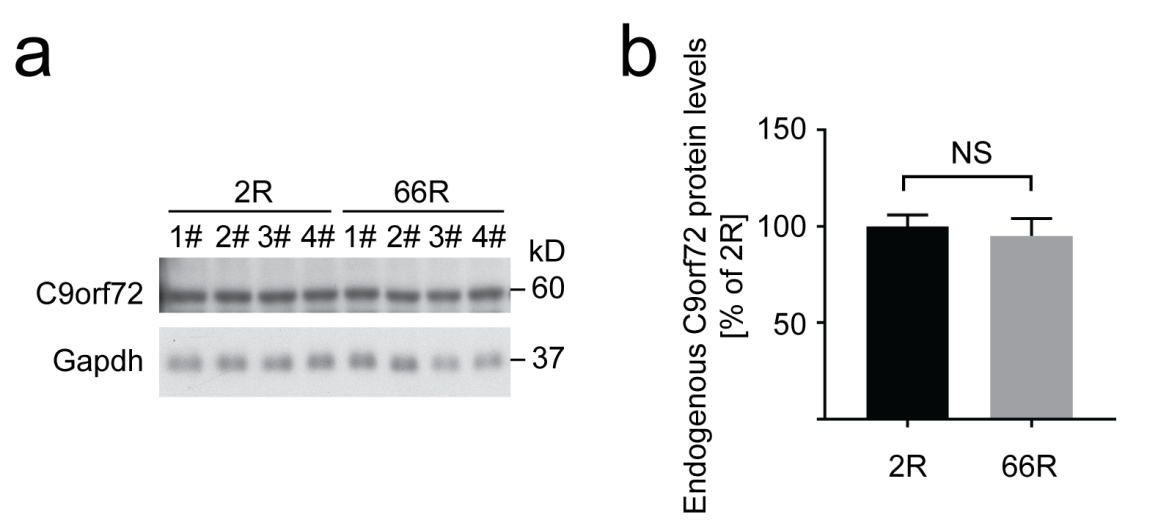


**Supplementary Figure 8.** **Endogenous C9orf72 protein levels in 2R- and 66R-injected mouse brain.** **(a)** Western blot of brain tissue obtained from 2R- or 66R-injected wild-type mice using an antibody against C9orf72. Gapdh was used as a loading control. **(f)** Protein quantification of endogenous C9orf72 protein levels in (a). The graph represents the mean ± S.E.M. by Student’s t-test; NS, not significant.

**Supplementary Figure 9**


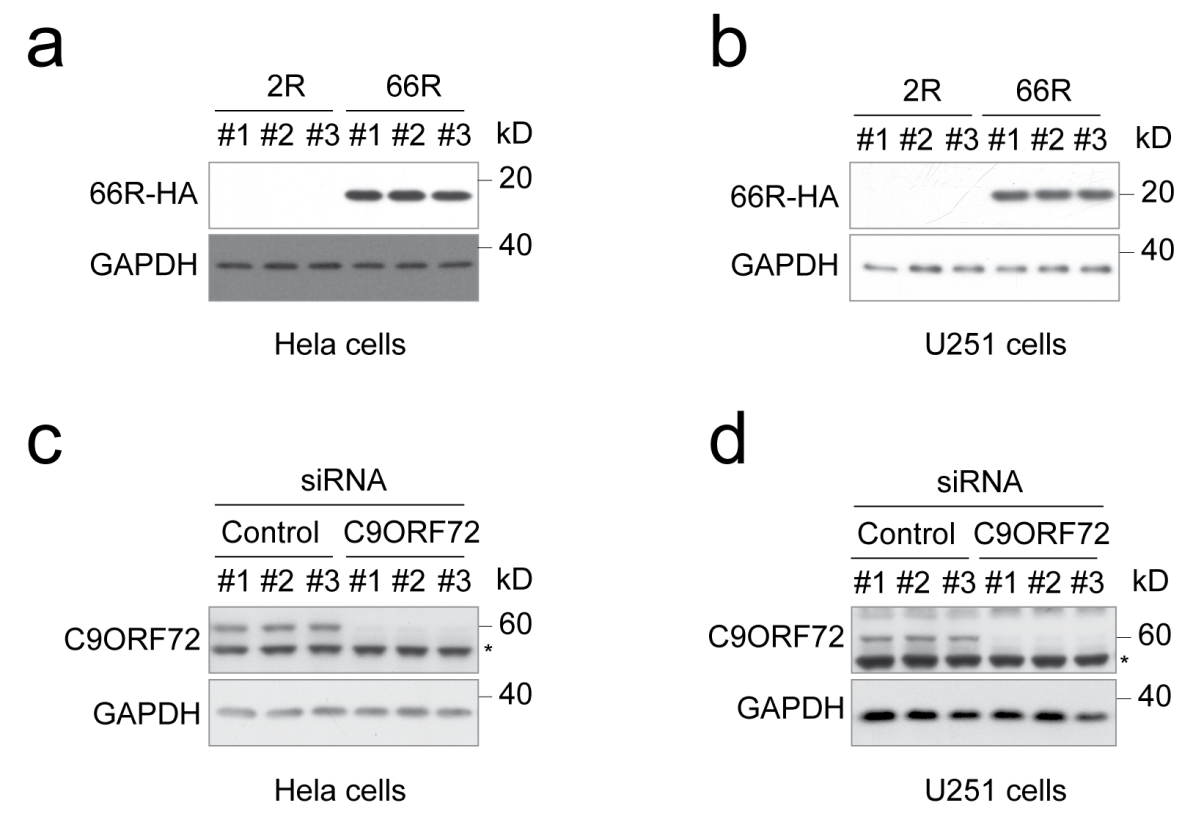


**Supplementary Figure 9.** Validation of (GGGGCC)_66_ repeat overexpression and *C9ORF72* knockdown. **(a-b)** Western blot of HeLa (a) and U251 (b) cells transfected with either 2R or 66R pAAV for 48 hrs. Repeat overexpression was confirmed by an antibody against the HA tag. **(c-d)** Western blot of HeLa (c) and U251 (d) cells transfected with control siRNA or an siRNA against *C9ORF72* for 72h. Non-specific bands are indicated (*) and GAPDH was used as a loading control.

**Supplementary Figure 10**


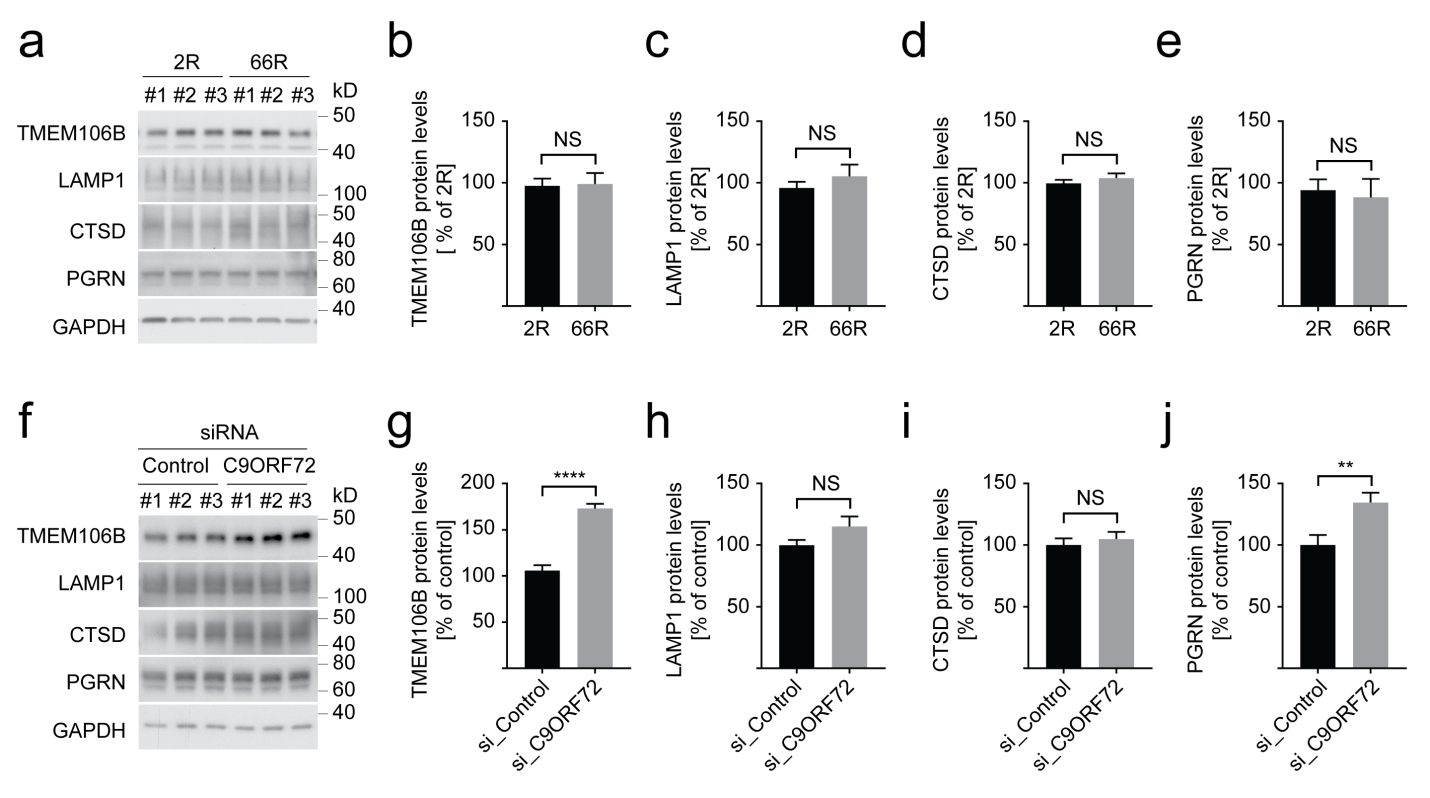


**Supplementary Figure 10.** **The effect of (GGGGCC)_66_ overexpression or *C9ORF72* knockdown on TMEM106B protein levels in U251 cells.** **(a)** Representative Western blot of U251 cells transfected with either 2R or 66R pAAV. **(b-e)** Protein quantification of TMEM106B (b), LAMP1 (c), CTSD (d), and PGRN (e) in cells transfected as described in (a). **(f)** Representative Western blot of U251 cells transfected with either control siRNA or siRNA against *C9ORF72*. **(g-j)** Protein quantification of TMEM106B (g), LAMP1 (h), CTSD (i), and PGRN (j) in cells transfected as described in (f). GAPDH was used as the loading control. Graphs represent the mean ± S.E.M. by Student’s t-test, and n=9 for all experimental groups. NS, not significant; **p<0.01, ****p<0.0001.
